# Peripheral self-reactivity regulates antigen-specific CD8 T-cell responses and cell division under physiological conditions

**DOI:** 10.1098/rsob.160293

**Published:** 2016-11-23

**Authors:** Lee Kim Swee, Zhen Wei Tan, Anna Sanecka, Nagisa Yoshida, Harshil Patel, Gijsbert Grotenbreg, Eva-Maria Frickel, Hidde L. Ploegh

**Affiliations:** 1 Whitehead Institute for Biomedical Research, Cambridge, MA 02142, USA; 2 Department of Microbiology, National University of Singapore, Singapore; 3 Department of Biological Sciences, Immunology Programme, National University of Singapore, Singapore; 4 Host-Toxoplasma Interaction Laboratory, The Francis Crick Institute, 1 Midland Road, London NW1 1AT, UK; 5 Bioinformatics and Biostatistics, The Francis Crick Institute, 1 Midland Road, London NW1 1AT, UK

**Keywords:** CD8 T cells, self-affinity, cell cycle

## Abstract

T-cell identity is established by the expression of a clonotypic T-cell receptor (TCR), generated by somatic rearrangement of TCRα and β genes. The properties of the TCR determine both the degree of self-reactivity and the repertoire of antigens that can be recognized. For CD8 T cells, the relationship between TCR identity—hence reactivity to self—and effector function(s) remains to be fully understood and has rarely been explored outside of the H-2^b^ haplotype. We measured the affinity of three structurally distinct CD8 T-cell-derived TCRs that recognize the identical H-2 L^d^-restricted epitope, derived from the Rop7 protein of *Toxoplasma gondii*. We used CD8 T cells obtained from mice generated by somatic cell nuclear transfer as the closest approximation of primary T cells with physiological TCR rearrangements and TCR expression levels. First, we demonstrate the common occurrence of secondary rearrangements in endogenously rearranged loci. Furthermore, we characterized and compared the response of Rop7-specific CD8 T-cell clones upon *Toxoplasma gondii* infection as well as effector function and TCR signalling upon antigenic stimulation *in vitro*. Antigen-independent TCR cross-linking *in vitro* uncovered profound intrinsic differences in the effector functions between T-cell clones. Finally, by assessing the degree of self-reactivity and comparing the transcriptomes of naive Rop7 CD8 T cells, we show that lower self-reactivity correlates with lower effector capacity, whereas higher self-reactivity is associated with enhanced effector function as well as cell cycle entry under physiological conditions. Altogether, our data show that potential effector functions and basal proliferation of CD8 T cells are set by self-reactivity thresholds.

## 1. Introduction

T cells play a key role in the immune response by assisting B cells in antibody production, activation of phagocytic cells or killing of infected cells. The stochastic assembly of an MHC-restricted αβ T-cell receptor (TCR) during thymic development [1] requires positive and negative selection steps that ensure minimal affinity for the complex of self-peptide and MHC molecules, without causing overt and deleterious response against self [2–5]. Survival of mature naive T cells in the periphery likewise relies on constant tonic signalling through the TCR, which of necessity involves self-MHC–peptide combinations [6].

Activation of antigen-specific T cells leads to clonal expansion and—in the case of CD4 T cells—functional polarization [7]. The characterization of Th1 and Th2 CD4 cells has been expanded upon by the description of additional CD4 T-cell subsets [8–11]. Each of these subsets occupies a functional niche during the immune response, allowing countermeasures tailored to a variety of pathogens. Furthermore, investigation of the variegated expression of nine different phenotypic markers, cytokines and cytotoxic proteins, has suggested considerable functional diversity in CD8 T-cell response [12]. Unlike CD4 T cells, this diversity might represent a continuum of distributed properties, rather than truly discrete CD8 T-cell subsets. The full extent of heterogeneity of effector CD8 T cells and the functional mechanism(s) that underlie it remain to be uncovered.

For CD8 T cells, the affinity of the TCR for the antigen–MHC complex dictates the extent of the primary and memory immune response [13]. CD8 T cells can execute their effector functions even upon *in vivo* stimulation with ligands that are relatively poor agonists [13]. For a given CD8 T-cell clone, the strength of TCR ligation positively correlates with IFNγ production, proliferation and memory formation [13]. Whether differences in TCR affinity for antigen between CD8 T-cell clones of identical specificity necessarily correlate with their respective effector functions remains to be investigated.

All αβ T cells require continuous interactions of the TCR with a complex set of self-peptide–MHC complexes not only in the course of development, but also in the periphery to ensure their survival. Affinity for self-peptide–MHC is intrinsic to each T-cell clone. Consequently, the intensity of such tonic signalling will presumably leave an imprint that may influence T-cell function upon TCR ligation with a foreign peptide–MHC complex. Levels of CD5 expression correlate with TCR self-reactivity for self-peptide MHC [14–16]. Recent studies have established a correlation between self-reactivity and T-cell effector functions, although with some contradictory findings [15–18]. Possible mechanisms underlying functional differences between CD5^low^ and CD5^high^ T cells include enhanced basal TCR signalling, as inferred from increased CD3ζ phosphorylation at rest [15,17], or greater sensitivity to inflammatory signals [16].

There are no comparisons for CD8 T-cell clones that share the same TCR specificity to explore whether the affinity of the TCR for antigen–MHC and/or affinity for self correlates with effector functions. It also remains to be determined whether there are functional differences between CD8 T-cell clones equipped with TCRs of similar specificity and, if so, what factors shape such differences. Here, we measured the affinity of the TCR for antigen–MHC for CD8 T cells from three different lines of transnuclear (TN) mice, all of which recognize the identical epitope, derived from the Rop7 protein of *Toxoplasma gondii* in complex with H-2 L^d^ [19]. We characterized Rop7 CD8 T-cell activation *in vivo* upon *Toxoplasma gondii* infection as well as antigen-dependent and -independent stimulation *in vitro*. Our data highlight major intrinsic differences in effector capacity between Rop7 clones regardless of the stimulation. We also demonstrate that self-reactivity thresholds determine effector functions: lower self-reactivity correlated with decreased functionality, whereas increased self-reactivity was associated with enhanced effector functions. Transcriptome-wide analysis of Rop7 CD8 T-cell clones showed major differences in expression of cell cycle-associated genes. In this manner, the identity of the clonotypic TCR contributes to the function of T cells beyond antigen recognition, by setting the degree of self-reactivity through basal TCR signalling as well as through mitotic status at rest and prior to activation.

## 2. Results

### 2.1. T-cell development and endogenous T-cell receptor rearrangements in Rop7-I, -II and -III transnuclear mice

**Figure 1.**
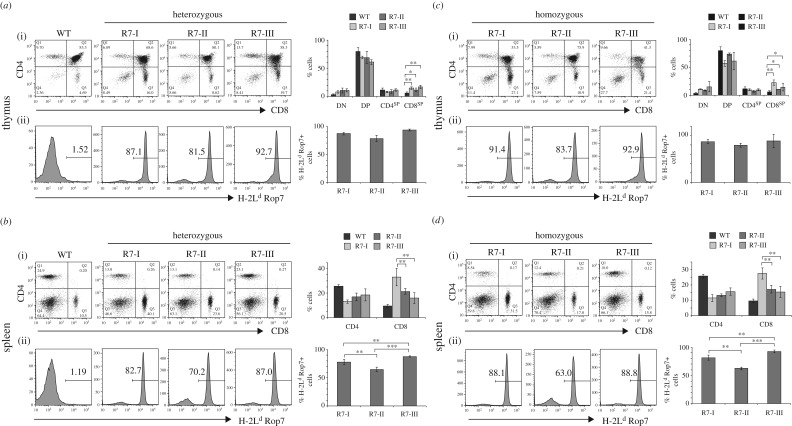
T-cell development in Rop7-I, -II and -III homozygous or heterozygous for TCRα and β. (*a*) (i) Dot plots show CD4 versus CD8 staining on thymocytes from WT, Rop7-I, -II or -III mice heterozygous for a Rop7-specific TCRα and β chain. (ii) Histograms show H-2 L^d^-Rop7 staining gated on CD8 single-positive (CD8^SP^) thymocytes. Upper bar histograms show mean percentage of thymocyte subsets. Error bars: standard deviation (*n* = 3–5). Lower histograms show mean percentage of H-2 L^d^-Rop7 cells within CD8^SP^ cells. Error bars: standard deviation (*n* = 3–5). (*b*) (i) Dot plots show CD4 versus CD8 staining on splenocytes from WT, Rop7-I, -II or -III mice heterozygous for an H-2 L^d^-Rop7-specific TCRα and β chain. (ii) Histograms show H-2 L^d^-Rop7 staining gated on CD8^+^ splenocytes. Upper bar histograms show mean percentage of CD4 and CD8 subsets. Error bars: standard deviation (*n* = 3–5). Lower histograms show mean percentage of H-2 L^d^-Rop7 cells within CD8 cells. Error bars: standard deviation (*n* = 3–5). (*c*) (i) Dot plots show CD4 versus CD8 staining on thymocytes from Rop7-I, -II or -III mice homozygous for a Rop7-specific TCRα and β chain. (ii) Histograms show H-2 L^d^-Rop7 staining gated on CD8 single positive (CD8^SP^) thymocytes. Upper bar histograms show mean percentage of thymocytes subsets. Error bars: standard deviation (*n* = 3–4). Lower histograms show mean percentage of H-2 L^d^-Rop7 cells within CD8^SP^ cells. Error bars: standard deviation (*n* = 3–4). (*d*) (i) Dot plots show CD4 versus CD8 staining on splenocytes from Rop7-I, -II or -III mice homozygous for a Rop7-specific TCRα and β chain. (ii) Histograms show H-2 L^d^-Rop7 staining gated on CD8^+^ splenocytes. Upper bar histograms show mean percentage of CD4 and CD8 subsets. Error bars: standard deviation (*n* = 3–4). Lower histograms show mean percentage of H-2 L^d^-Rop7 cells within CD8 cells. Error bars: standard deviation (*n* = 3 to 4). *p < 0.05, **p < 0.01, ***p < 0.001 (Student's *t*-test).

We reported the generation, using somatic cell nuclear transfer (SCNT) [20–22], of three lines of CD8 T-cell TN mice, all of which bear an αβ TCR specific for the peptide IPAAAGRFF derived from the Rop7 protein of *Toxoplasma gondii* [19]. The CD8 T cells that served as SCNT donors were obtained by cell sorting, using H-2 L^d^ tetramers loaded with the Rop7 epitope. We refer to these lines of mice as Rop7-I, -II and -III (R7-I, -II and -III in figures). Thymocyte development in Rop7-I, -II and -III mice heterozygous for the TN TCRα and β chain progressed normally, with a slight increase in CD8 single-positive cells (CD8^SP^) due to the expression of the class I MHC-restricted TCR (figure 1*a*(i)). The majority but not all CD8^SP^ cells expressed a Rop7-specific TCR (figure 1*a*(ii)). We observed a skewed ratio of mature CD8 T cells in the spleen of all Rop7 mice compared to WT mice and significant differences in the percentage of CD8 T cells between Rop7-I versus Rop7-II and -III (figure 1*b*). The percentage of CD8 T cells that expressed a Rop7-specific TCR was different for Rop7-II versus Rop7-I and -III CD8 cells. Indeed, whereas the fraction of tetramer-negative cells was moderate in Rop7-I and -III mice, up to 30% of CD8 T cells did not stain for H2-L^d^ Rop7 in Rop7-II mice (figure 1*b*). This could be due to rearrangements of a TCRα V-J combination on the endogenous germline allele or to secondary rearrangements at the original TN TCRα locus. To distinguish between both possibilities, we bred Rop7 mice to homozygosity for the TN TCRα and β chain. Thymocyte development from double-negative to single-positive cells in TCRα and β homozygous mice occurred was comparable to T-cell development observed in the respective heterozygous mice (figure 1*c*). Most importantly, we observed not only the presence of CD8^+^ tetramer-negative cells in all Rop7 mice, although at different percentages, but also CD4 T cells in readily detectable numbers (figure 1*c,d*), all of which were tetramer-negative (electronic supplementary material, figure S1). Both findings demonstrate the occurrence of secondary rearrangements at the TCRα, possibly TCRβ locus. The percentages of mature H2-L^d^ Rop7^+^ CD8 T cells were different for the different Rop7 mice but altogether similar to percentages in the heterozygotes (figure 1*d*). Altogether, T-cell development occurred normally in Rop7 mice. Furthermore, our data show that secondary TCR rearrangements are common for the TN rearranged loci.

### 2.2. Rop7-I, -II and -III T-cell receptor recognize Rop7 cognate antigen in a quantitatively and qualitatively different manner

**Figure 2.**
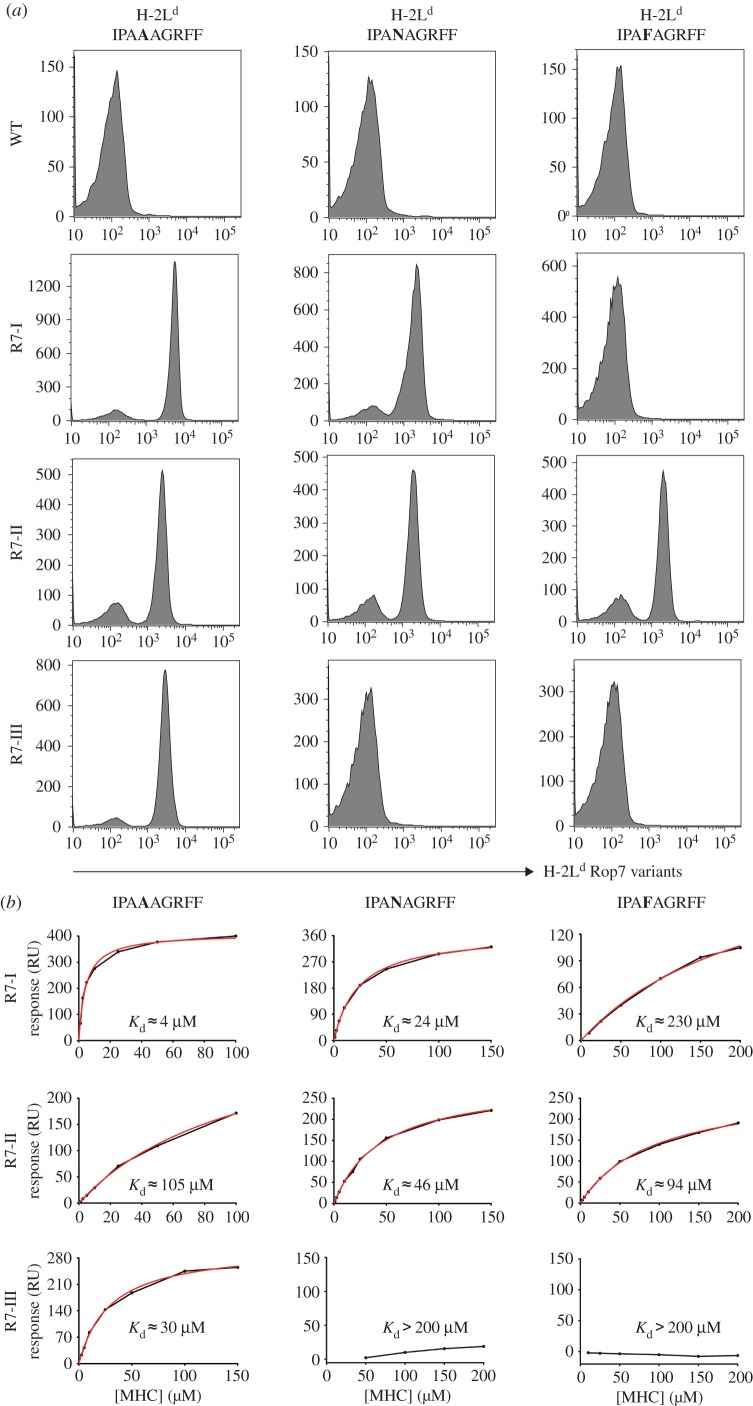
Binding of Rop7-I, -II and -III to Rop7 peptide variants. (*a*) Splenocytes from WT or Rop7-I, -II and -III heterozygous mice were stained for CD8 and with H-2 L^d^ tetramers loaded with the indicated peptides. Histograms show H-2 L^d^-Rop7 variants staining on CD8 T cells from WT or Rop7 mice. (*b*) TCRs from Rop7-I, -II and -III T cells were recombinantly expressed and refolded. Binding affinities for IPAAAGRFF-, IPAFAGRFF- and IPANAGRFF-loaded H-2 L^d^ were measured with a BIAcore 3000 and analysed using an equilibrium model. The raw data (black lines), fits (red lines) and calculated dissociation constant are shown in each panel.

The TCRs of the three Rop7 lines comprise distinct V, (D) and J elements for their rearranged TCR α and β genes, resulting in CDR3 regions unrelated in sequence, suggesting differences in the details of recognition of the Rop7-H-2 L^d^ complex [19]. We already noted subtle differences in dissociation rates of their TCRs from Rop7-H-2 L^d^ tetramers [19]. To determine whether the TCRs used by CD8 T cells from Rop7-I, -II and -III mice recognize the Rop7 epitope in a qualitatively different manner, we designed a set of altered peptide ligands and monitored the ability of CD8 T cells to recognize such ligands in the context of H-2 L^d^, using the caged tetramer approach [23–25]. Indeed, single amino acid substitutions differentially affected tetramer binding to CD8 T cells from Rop7-I, -II and -III (figure 2*a*). CD8 T cells from all three lines of Rop7 mice bound tetramers loaded with the nominal antigen (IPAAAGRFF), with little quantitative variation. Binding of H-2 L^d^-IPAAAGRFF to Rop7-II T cells was slightly less avid than binding to Rop7-I or -III, suggesting a lower affinity. Rop7-I and -II, but not Rop7-III T cells bound IPA**N**AGRFF-loaded tetramers, whereas only Rop7-II T cells bound IPA**F**AGRFF-loaded tetramers. We expressed recombinant Rop7-I, -II and -III TCRs to measure their affinity for epitope-loaded H-2 L^d^ molecules by surface plasmon resonance (figure 2*b*). The calculated dissociation constants of Rop7-I, -II and -III TCRs for H-2 L^d^-IPAAAGRFF were 4 µM, 105 µM and 30 µM, respectively (figure 2*b*). Rop7-I TCR had greater affinity for H-2 L^d^-IPA**N**AGRFF compared with the Rop7-II TCR (figure 2*b*, Rop7-I: 24 µM versus Rop7-II: 46 µM), whereas Rop7-II TCR had greater affinity for H-2 L^d^-IPA**F**AGRFF (figure 2*b*, Rop7-I: 230 µM versus Rop7-II: 94 µM). We conclude that each of the Rop7-specific CD8 T cells recognizes the epitope-H-2 L^d^ complex differently, as established by this substitution analysis.

### 2.3. Proliferation and activation of Rop7-I, -II and -III T cells upon infection with *Toxoplasma gondii*

**Figure 3.**
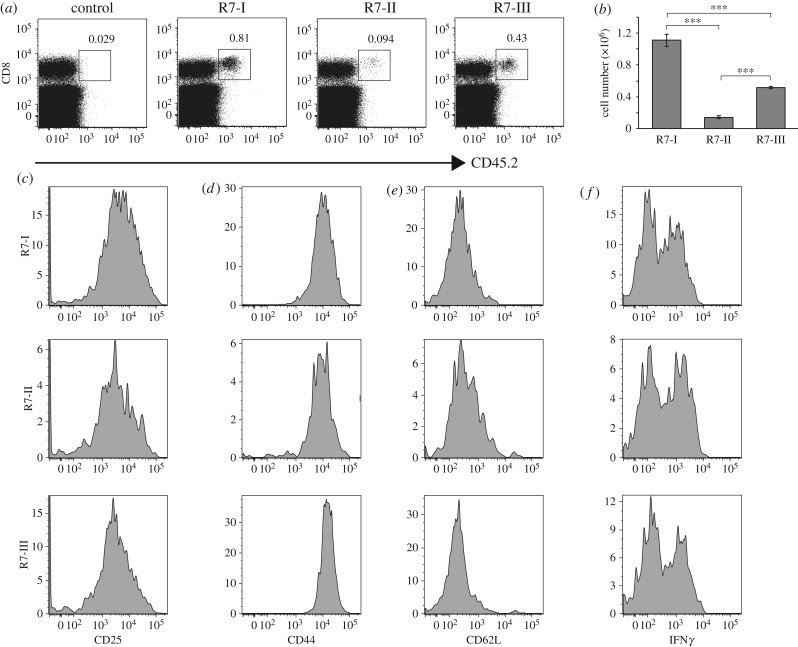
Rop7-I, -II and -III CD8 T cell expansion and phenotype after *Toxoplasma gondii* infection. A measure of 1 × 10^5^ CD8^+^ tetramer^+^ sorted T cells from Rop7 -I, -II or -III heterozygous mice were transferred intravenously into CD45.1 congenic BALB/c mice. Twenty-four hours after T-cell transfer, mice were infected with 2 × 10^4^ *Toxoplasma gondii* tachyzoites. (*a*) Dot plots show the percentage of CD8^+^ CD45.2^+^ donor cells in the spleen at day 9 after infection. (*b*) Histograms show the total cell number of transferred Rop7 T cells in the spleen of recipient mice at day 9 after infection. Error bars: standard deviation (*n* = 3). (*c–e*) Histograms show CD25, CD44 and CD62 L expression on transferred Rop7 T cells in the spleen at day 9 after infection respectively. (*f*) Histograms show IFNγ expression by transferred Rop7 T cells from the spleen of recipient mice at day 9 after infection stimulated *in vitro* with Rop7 peptide. ***p < 0.001 (Student's *t*-test).

To explore whether Rop7 CD8 T cells were equally capable of participating in an immune response, we transferred 1 × 10^5^ sorted CD8^+^ tetramer^+^ T cells from Rop7-I, -II or -III mice into congenic BALB/c CD45.1 mice and monitored their expansion upon infection with *Toxoplasma gondii*. Cells from Rop7-I and -III mice had proliferated at day 9 after infection (figure 3*a,b*). By contrast, Rop7-II CD8 T cells expanded less in response to *Toxoplasma* (figure 3*a,b*). Nevertheless, Rop7-II T cells expressed similar proportions of CD25, CD44 and CD62 L compared to Rop7-I and -III T cells, suggesting that all T cells were antigen-experienced and activated (figure 3*c–e*). Rop7-II T cells produced similar amounts of IFNγ compared to Rop7-I and -III T cells upon *in vitro* re-stimulation (figure 3*f*). Unlike our experience with T57-specific CD8 T cells [19], the transfer of Rop7 T cells specific for the *Toxoplasma* late antigen Rop7 [23] had no appreciable impact on pathogen load at day 9 after infection (electronic supplementary material, figure S2).

### 2.4. Rop7-I, -II and -III CD8 T-cell activation upon antigenic stimulation *in vitro*

**Figure 4.**
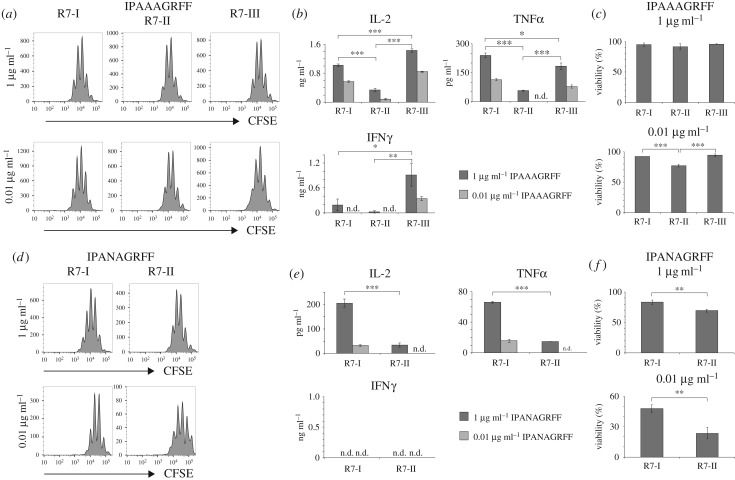
Rop7 CD8 T cells proliferation and cytokine secretion upon Rop7 peptide variant stimulation. Sorted CD8^+^ tetramer^+^ T cells from pooled lymph nodes and spleen of Rop7-I, -II or -III heterozygous mice were labelled with CFSE and stimulated with BMDC loaded with Rop7 peptide variants at indicated concentrations. (*a*) Histograms show CFSE dilution on sorted CD8 T cells from Rop7-I, -II or -III mice stimulated with IPAAAGRFF peptide-loaded BMDC after 3 days of stimulation. (*b*) IL-2, TNFα and IFNγ concentration measured in culture supernatant after 3 days of stimulation. Mean of three technical replicates (T-cell stimulation). Error bars: standard deviation (*n* = 3). Representative of three independent experiments. (*c*) Bar histograms show the percentage of living cells (Zombie Aqua negative) after 3 days of stimulation with IPAAAGRFF-loaded BMDC. Mean of three technical replicates (T-cell stimulation). Error bars: standard deviation (*n* = 3). Representative of three independent experiments. (*d*) Histograms show CFSE dilution on sorted CD8 T cells from Rop7-I or -II heterozygous mice stimulated with IPANAGRFF peptide-loaded BMDC after 3 days of stimulation. (*e*) IL-2, TNFα and IFNγ concentration measured in culture supernatant after 3 days of stimulation. Mean of three technical replicates (T-cell stimulation). Error bars: standard deviation (*n* = 3). Representative of three independent experiments. (*f*) Bar histograms show the percentage of living cells (Zombie Aqua negative) after 3 days of stimulation with IPANAGRFF-loaded BMDC. *p < 0.05, **p < 0.01, ***p < 0.001 (Student's *t*-test).

The decreased ability of Rop7-II cells to robustly proliferate upon *Toxoplasma* infection might be due to several cell-intrinsic or cell-extrinsic factors that are challenging to investigate in the context of an infection. To compare the function of Rop7 CD8 T cell upon antigenic stimulation under defined conditions, we incubated sorted CD8^+^ H-2 L^d^-Rop7^+^ cells with bone marrow-derived dendritic cells (BMDC) loaded with different amounts of IPAAAGRFF peptide. Rop7-I, -II and -III T cells were able to proliferate upon antigenic stimulation in a dose-dependent manner (figure 4*a*). We observed sizable differences in the production of cytokines upon stimulation. Rop7-II CD8 T cells produced overall less IL-2, TNFα and IFNγ, whereas Rop7-III CD8 T cells secreted increased amounts of IL-2 and IFNγ but not TNFα when compared with Rop7-I (figure 4*b*). By contrast, all Rop7 CD8 T cells were cytotoxic, albeit with subtle but notable differences (electronic supplementary material, figure S3). Differences in *in vivo* expansion upon infection (figure 3*a,b*) might be due to T-cell apoptosis caused by insufficient or too much stimulation. To investigate how antigen affinity and therefore signal strength might influence T-cell survival following activation, we monitored cell viability following stimulation. Rop7-II T cells showed a significantly increased tendency to cell death at a lower degree of stimulation (figure 4*c*). The decreased ability to secrete cytokines and worse survival of Rop7-II cells might be a cell-intrinsic feature or due to a lower affinity for IPAAAGRFF peptide. As TCR of Rop7-I and -II CD8 T cells has comparable affinity for IPA**N**AGRFF peptide (figure 2), we stimulated Rop7-I and -II CD8 T cells with BMDC loaded with different doses of IPA**N**AGRFF peptide. Both Rop7-I and -II CD8 T cells were able to proliferate upon stimulation (figure 4*d*). Nevertheless, Rop7-II T cells produced significantly fewer cytokines (figure 4*e*) and were more prone to cell death at both peptide concentrations further suggesting this is an intrinsic trait (figure 4*f*). Our data show that upon antigenic stimulation, Rop7-III CD8 T cells secreted more IL-2 and IFNγ but not TNFα than Rop7-I, whereas Rop7-II produced fewer cytokines and were more prone to dying, regardless of the peptide used for stimulation.

### 2.5. Differences in T-cell receptor signalling in CD8 T cells from Rop7 mice

**Figure 5.**
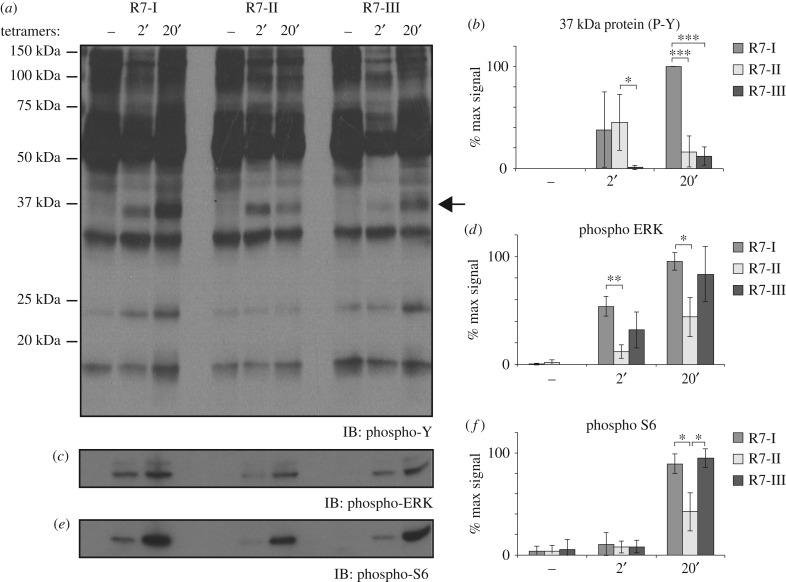
TCR signalling in CD8 T cells from Rop7-I, -II and -III mice. Untouched MACS-purified CD8 T cells from pooled spleen and lymph nodes of Rop7-I, -II and -III homozygous mice were stimulated with H-2 L^d^-Rop7 tetramers for 2 or 20 min. (*a*) Tyrosine residues, (*b*) ERK and (*c*) S6 phosphorylation was analysed in total lysates by SDS-PAGE and immunoblotting. (*d–e*) Quantification of band intensity for 37 kDa-size protein (arrow), phospho-ERK and phospho-S6. Average of three biological replicates. Error bars: standard deviation (*n* = 3). *p < 0.05, **p < 0.01, ***p < 0.001 (Student's *t*-test).

The antigenic stimulation of Rop7 CD8 T cells *in vitro* highlighted major differences in the outcome of activation (cytokine secretion and cell survival). To investigate whether observed differences are due to changes in TCR signalling upon antigen recognition for each Rop7 T-cell lines, we stimulated equal numbers of tetramer^+^ cells with H-2 L^d^-Rop7 for 2 or 20 min and followed protein phosphorylation by immunoblotting. We observed marked qualitative differences in the kinetics of tyrosine phosphorylation including differential phosphorylation of a 37 kDa protein, possibly LAT (figure 5*a,b*, arrow). Monitoring of the phosphorylation of both ERK (figure 5*c,d*) and S6 (figure 5*e,f*) revealed further decreased level of phosphorylation at 20 min in Rop7-II CD8 T cells compared with Rop7-I and -III T cells. In brief, these data demonstrate that TCR signalling occurs in a unique and distinct manner in all three Rop7 CD8 T cells.

### 2.6. Antigen-independent stimulation reveals differences in Rop7 CD8 T-cell-intrinsic properties

**Figure 6.**
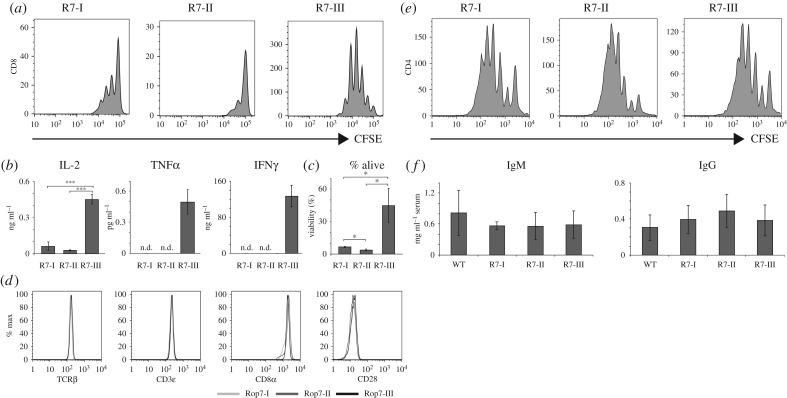
Non-specific stimulation of CD8 T cells from Rop7-I, -II and -III mice. (*a,b*) Sorted CD8^+^ H-2 L^d^-Rop7^+^ T cells from pooled lymph nodes and spleen of Rop7-I, -II or -III heterozygous mice were labelled with CFSE and stimulated with plate-bound anti-CD3 and anti-CD28. (*a*) Histograms show CSFE dilution after 3 days of stimulation. (*b*) IL-2, TNFα and IFNγ concentration measured in culture supernatant after 3 days of stimulation. Mean of three technical replicates (T-cell stimulation). Error bars: standard deviation (*n* = 3). Representative of three independent experiments. (*c*) Bar histograms show the percentage of living cells (Zombie Aqua negative) after 3 days of stimulation with anti-CD3 and anti-CD28. Mean of three technical replicates. Error bars: standard deviation (*n* = 3). Representative of three independent experiments. (*d*) Histograms show the expression of TCRβ, CD3ɛ, CD8α and CD28 on CD8^+^ H-2 L^d^-Rop7^+^ T cells from the spleen of Rop7-I, -II or -III mice. (*e*) CD4^+^ cells from pooled lymph nodes and spleens of Rop7-I, -II or -III heterozygote mice were labelled with CFSE and stimulated with plate-bound anti-CD3 and anti-CD28. Histograms show T-cell proliferation as measured by CFSE dilution after 5 days of stimulation. Representative of two independent experiments. (*f*) IgM and IgG antibody titres in the serum of WT, Rop7-I, -II or -III mice. Error bars: standard deviation (*n* = 3). *p < 0.05, **p < 0.01, ***p < 0.001 (Student's *t*-test).

*In vivo* and *in vitro* antigenic stimulation demonstrate underlying differences between Rop7-I and -III CD8 T cells, and overall decreased effector functions for Rop7-II CD8 T cells. To distinguish between the effect of antigen affinity and cell-intrinsic properties, we stimulated Rop7 CD8 T cells with plate-bound anti-CD3 and anti-CD28. Upon activation, Rop7-III CD8 T cells proliferated strongly. By contrast, Rop7-I CD8 T cells proliferated moderately, whereas Rop7-II T cells barely proliferated at all (figure 6*a*). Rop7-III CD8 T cells secreted IL-2, TNFα and IFNγ, whereas Rop7-I and -II T cells produced an only just detectable amount of IL-2 (figure 6*b*). Moreover, we observed drastic differences in survival of stimulated T cells that correlated with proliferation (figure 6*c*). There were no differences in the levels of expression of TCRβ, CD3ε, CD8α and CD28 among the different naive Rop7 CD8 T cells (figure 6*d*). Thus, observed differences cannot be due to changes in TCR complex expression. In order to exclude non-specific defects in lymphocyte biology of Rop7 mice, we measured proliferation of sorted CD4 T cells, as well as IgM (T-independent) and IgG (T-dependent) titres in Rop7 mice. There were no differences in the ability of CD4 cells to proliferate upon anti-CD3/anti-CD28 stimulation (figure 6*e*). Likewise, IgM and IgG titers were similar for the various Rop7 and WT mice. Altogether, our results show intrinsic differences in effector function of Rop7 CD8 T cells upon antigen-independent CD3/CD28 stimulation in the following hierarchy in terms of strength of response: Rop7-III > Rop7-I > Rop7-II. Importantly, those differences were specific to CD8 T cells as CD4 T-cell proliferation and antibody secretion was indistinguishable for the different Rop7 mice.

### 2.7. The response of Rop7-II T cells correlates with decreased affinity for self-antigen(s)

The integration of signals received during thymic selection and through tonic engagement of the TCR in the periphery may set the responsiveness of mature CD8 T cells [15,17]. For example, T cells that barely pass the criteria for positive selection in the thymus may put in place qualitatively different signalling machinery to ensure peripheral survival, in a manner distinct from T cells that are close to the threshold for thymic deletion. The latter type of T cell presumably experiences signals of a different quality in the periphery as well, with possible consequences for the specification of function.

**Figure 7.**
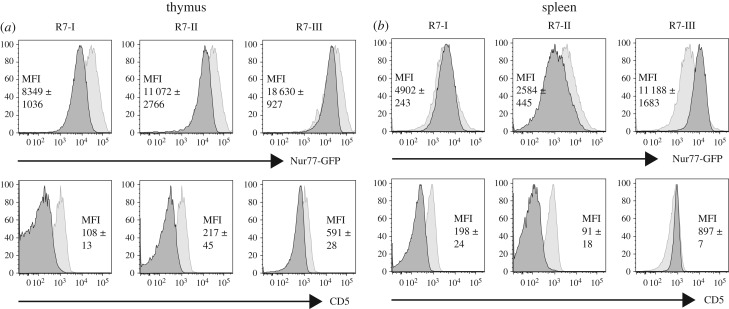
Relative self-reactivity of Rop7-I, -II and -III CD8 T cells. Rop7-I, -II and -III mice were crossed to Nur77-GFP transgenic mice. (*a*) Histograms show (i) GFP or (ii) CD5 levels on CD8^SP^ H-2 L^d^-Rop7^+^ T cells (dark grey histograms) from mice heterozygote for TCRα and β compared to CD8^SP^ cells from a control Nur77-GFP transgenic mouse (light grey histograms). MFI +/− standard deviation of two independent values. Representative of at least five mice per genotype. (*b*) Histograms show (i) GFP or (ii) CD5 level on CD8^+^ tetramer^+^ Rop7 T cells (dark grey histograms) compared to CD8^+^ cells from a control Nur77-GFP transgenic mouse (light grey histograms).

To investigate whether the phenotype of Rop7-II T cells correlates with differences in tonic signalling (affinity for self-peptide–MHC complexes), we crossed Rop7 TN mice with Nur77-GFP BAC transgenic mice [26]. In these mice, the level of GFP expression correlates with the strength of TCR signalling, which, in the absence of the nominal peptide antigen, depends on endogenous peptide–MHC complexes, and is therefore a proxy for self-reactivity, together with the expression level of CD5 [14]. Thymic CD8^SP^ cells from both Rop7-I and -II mice expressed lower levels of Nur77-GFP and CD5 compared with CD8^SP^ cells from WT or Rop7-III mice (figure 7*a*). Mature Rop7-II T cells expressed less Nur77-GFP than polyclonal CD8 T cells and than Rop7-I and -III T cells (figure 7*b*). Moreover, Rop7-II expressed less CD5 compared with polyclonal CD8 T cells (figure 7*b*). Rop7-III T cells had increased levels of Nur77-GFP when compared with Rop7-I, as well as increased CD5 levels (figure 7*b*). Altogether, reduced cytokine production and proliferation of Rop7-II T cells correlate with altered distal signalling and decreased self-reactivity.

### 2.8. Increased self-affinity is associated with cell proliferation under physiological conditions

**Figure 8.**
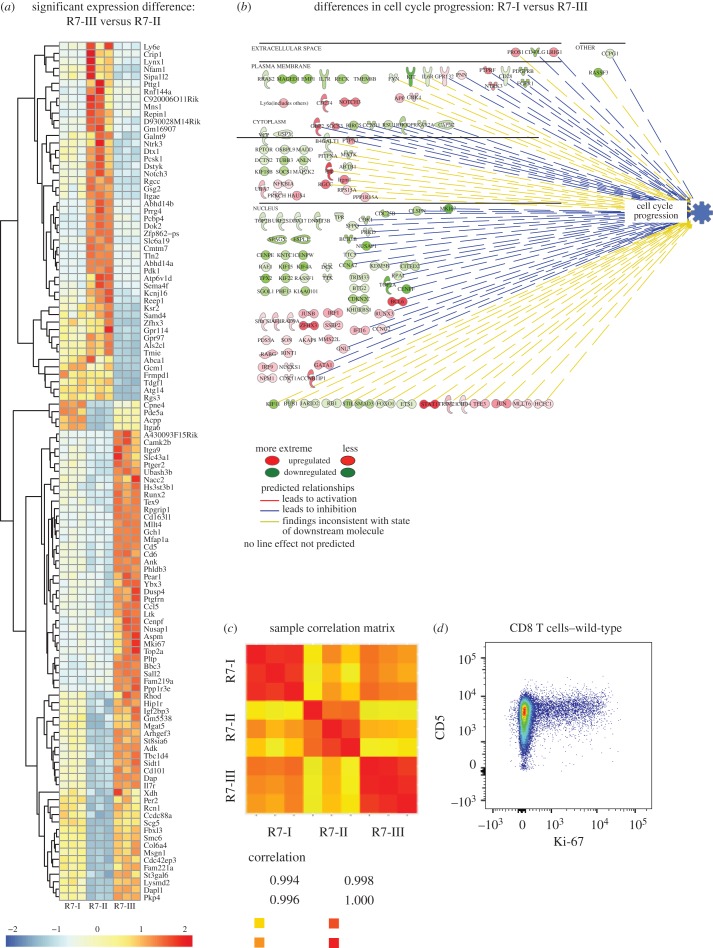
Transcriptional profile of non-activated R7-I, R7-II and R7-III CD8 T cells. (*a*) Row centred heatmap of transcripts per million (TPM) values for genes with a significant expression difference of over twofold between R7-III and R7-II homozygous CD8 T cells. (*b*) Ingenuity pathway of genes associated with cell cycle progression and found to be significantly differentially expressed between R7-I and R7-III homozygous CD8 T cells. (*c*) Sample correlation matrix for R7-I, R7-II and R7-III CD8 T cells generated from TPM values for expressed genes (assumed to have a minimum TPM of 1 across all samples in the experiment). Each row and column represents a replicate biological sample of the denoted Rop7 clones. (*d*) CD5 versus Ki-67 expression gated on CD8 T cells from WT BALB/c mice.

In order to investigate mechanisms that may account for differences in effector functions of Rop7 CD8 T cells at steady state, we performed a transcriptome-wide analysis of unstimulated Rop7 CD8 T cells. As Rop7-III T cells had the highest affinity for self-antigen and Rop7-II the lowest (figure 7*b*), the transcriptional profile of Rop7-III was compared with that of Rop7-II. One hundred and fifteen genes were identified to be significantly expressed with at least a twofold difference (figure 8*a*; electronic supplementary material, table S1). Gene ontology analysis between these two Rop7 lines uncovered major differences in cell-cycle and proliferation-related pathways (electronic supplementary material, table S2). As expected, based on differential TCR–MHC binding strength between Rop7-III and -II, we also observed differences in expression of genes related to TCR signalling (electronic supplementary material, table S2). In the unstimulated state, Rop7-I showed a less active signature than Rop7-III for genes associated with cell cycle progression (figure 8*b*). In this regard, Rop7-II CD8 T cells are more similar to Rop7-I than to Rop7-III (electronic supplementary material, figure S4). A global comparison of expressed transcripts between the three unstimulated Rop7 CD8 T cells clones showed that Rop7-I and -III are more similar to each other than to Rop7-II (figure 8*c*). To investigate whether our findings could be extended to a polyclonal CD8 T-cell population, we monitored cell cycle status under physiological conditions through expression of the proliferation-associated protein Ki-67. Ki-67^+^ proliferating cells were almost exclusively found among CD5^high^ CD8 T cells (figure 8*d*). In conclusion, our transcriptomic analysis reveals that Rop7-III clone, like polyclonal CD5^high^ T cells, demonstrates enhanced proliferation or readiness for cell cycle entry, when compared with both Rop7-I and -II clones. Transcriptomic analysis showed Rop7-II to be clearly different from the other Rop7-specific CD8 T-cell clones, a trait that must be imposed by identity of its clonotypic TCR.

## 3. Discussion

We used three lines of mice, created by SCNT, as a source of clonotypically distinct, but homogeneous CD8 T cells, all of which recognize the identical Rop7-H-2 L^d^ peptide–MHC complex. A comparison of these CD8 T cells should allow us to assess the impact of the TCR's identity on the functional characteristics T cells as revealed also by the status of their transcriptome. This is important not only because it extends this type of analysis to an MHC haplotype other than H-2^b^, but also because no such comparison has been made for independently generated lines of mice bearing Class I MHC-restricted receptors of the same specificity on a genetically homogeneous background. We first analysed T-cell development in Rop7 mice heterozygous or homozygous for the TN TCRα and β chain and report the presence of appreciable percentage of CD8 cells that fail to stain with Rop7 tetramers, as well as the presence of a reduced, but important percentage of mature CD4 T cells. In heterozygous mice, the expression of a TCR other than the original TN receptor requires the endogenous rearrangement of the WT TCRα (or β) locus or secondary rearrangements at the TN TCRα (or β) locus. In homozygous mice however—because both alleles are rearranged—only productive secondary rearrangements at the TN TCRα (or β) locus can produce a TCR that is not specific for H-2 L^d^-Rop7. Therefore, our results demonstrate unambiguously that secondary rearrangements can occur at otherwise genetically unmanipulated loci and that these events are not rare considering the percentage of CD4 cells and tetramer^−^ CD8 T cells. The investigation of receptor editing and secondary rearrangement events have, until very recently, only been possible though knock-in of rearranged TCR segments in the α locus [27–29], a procedure bypassed by the TN approach in the present study.

We have measured the affinity of Rop7 TCR for H-2 L^d^-IPAAAGRFF antigen and its variants, and investigated the outcome of antigenic (Rop7 and variant) and (antigen-independent) anti-CD3/anti-CD28 stimulation. *In vitro* stimulation with IPAAAGRFF-loaded BMDC demonstrates that Rop7-III CD8 T cells produced significantly more IL-2 and IFNγ, but not TNFα, compared with Rop7-I CD8 T cells. Furthermore, Rop7-II cells secreted significantly fewer cytokines compared with Rop7-I and -III CD8 T cells and an increased proportion of cells died as the strength of stimulus decreased. Because the Rop7-II TCR has a significantly lower affinity for H-2 L^d^-IPAAAGRFF than the TCR of Rop7-I and -III (25- and 3-fold less, respectively), such differences could have been explained by differences in affinity. However, Rop7-II CD8 T cells showed an equally poor ability to secrete cytokines and enhanced cell death compared with Rop7-I CD8 T cells when stimulated with IPANAGRFF-loaded BMDC. In these conditions, the difference in affinity was less (approx. twofold), whereas differences in cytokine production remained large, suggesting inherent impairment of Rop7-II effector function. Stimulation of Rop7 CD8 T cells with anti-CD3/anti-CD28 (i.e. independently of antigen affinity) confirmed that Rop7-II CD8 T cells had lower intrinsic effector capacities as well as a marked propensity to cell death. Moreover, we observed differences between Rop7-I and -III T cells in proliferation, cytokine production and cell viability. Both inferior and superior effector functions might be intrinsically set by thresholds of self-reactivity. Indeed, for the CD8 clonal variants characterized here, self-reactivity positively correlates with effector capacities as demonstrated for several CD4 transgenic T cells [15,17] or a CD8 T cell polyclonal population [16]. In the context of a *Toxoplasma* infection, all three Rop7 CD8 T cells clonal variants proliferated and secreted cytokines. Nevertheless, we observed marked differences in the accumulation in the spleen and notably an increased number of Rop7-I versus -III (and -II) CD8 T cells. We propose the following hypothesis to explain differences between these *in vivo* and *in vitro* experiments. First, accumulation in the spleen might not faithfully reflect global clonal expansion, for example, due to changes in cell migration behaviour. Second, discrepancies might be explained by quantitative (antigen dose) and qualitative (cytokines, co-stimulation) differences in stimulation as well as other cell-extrinsic factors (e.g. nutrients) between both conditions. Third, although we have no indication of increased apoptosis, Rop7-III CD8 T cells may be unable to sustain long-lasting expansion.

Our analysis of protein phosphorylation upon antigenic stimulation has uncovered interesting differences in signal transduction as well. Although we observed a decreased level of ERK and S6 phosphorylation in Rop7-II T cells, differences between the various Rop7 T cells seemed qualitative rather than merely quantitative. In fact, accumulated data suggest that signalling might occur in a different manner for every single clone depending not only on signal strength, but also on cell state as determined by tonic signalling experienced in the periphery [15,17]. Differences in signal integration [30] are likely to explain heterogeneity in effector functions [12].

Our work and that of others [15–17] show a correlation between effector function of T cells and self-reactivity. How, then, does self-reactivity dictate future effector functions? Self-reactivity, hence tonic signalling, might impact the phospho-proteome of each T-cell clone in a different manner. Indeed, evidence of changes in protein phosphorylation at rest has been provided in two independent studies [15,17]. Self-reactivity might also imprint the trancriptome of each clone in physiological conditions. Data presented above as well as results from others [16] support this hypothesis. Our transcriptome-wide analysis showed that differences between unstimulated Rop7-II and -III CD8 T cells in cell cycle-associated genes were highly significant, even more so than TCR signalling-associated genes when considering adjusted *p*-values (electronic supplementary material, table S2). We confirmed these findings in a polyclonal CD8 T cell population. Indeed, the vast majority of cycling, Ki-67^+^ cells were CD5^high^. This correlation suggests a threshold of self-reactivity (and CD5 levels) beyond which cells enter the cell cycle under physiological conditions and in the absence of the Rop7 epitope. Homeostatic expansion, a process linked to CD5 expression level [31,32], is assumed to be driven by ‘self’ antigen when resources (e.g. IL-7) are abundant, as is the case in lymphopenia [33]. However, it is local rather than global cytokine excess that will be sensed by T cells. Of note, the IL-7 receptor (IL7r) gene itself was upregulated in Rop7-III T cells (figure 6*a*).

In conclusion, the concept of a ‘naive’ T cell as a fixed entity may need revision. Effector capacities of T-cell clones are shaped by the prior experience of self-reactivity. Future investigations focusing on signalling or transcriptomic analysis at the single-cell level in antigen-inexperienced T cells may very well uncover an even greater heterogeneity. In the very same way, the definition of ‘self’ in the context of these recent studies is worth reconsidering. ‘Homeostatic peptidome’ may be a more appropriate term than ‘genome-encoded self’.

## 4. Experimental procedures

### 4.1. Mice

BALB/c and BALB/c CD45.1 mice were purchased from the Jackson laboratory and bred in the animal facility of the Whitehead Institute for Biomedical Research (Cambridge, MA) or in the animal facility of the Francis Crick Institute, Mill Hill Laboratory (London, UK). Nur77-GFP transgenic mice were described in [26,34]. The generation of Rop7-I, -II and -III was described elsewhere [19]. Heterozygous and homozygous Rop7 mice were backcrossed for six generations or more on a BALB/c background.

### 4.2. Antibodies and reagents

Purified anti-CD3 (clone 17A2) and anti-CD28 (clone 37.51) antibodies, fluorescently labelled Granzyme B (clone 16G6), CD3ɛ (clone 145-2C11), CD4 (clone GK1.5), CD8 (clone 53-6.7), CD45.1 (clone A20) specific antibodies were purchased from eBioscience. Biotinylated anti-CD4 (clone GK1.5), anti-CD19 (clone 1D3), fluorescently labelled CD25 (clone 7D4), CD44 (clone 1M7), CD69 (clone H1.2F3), TNFα (clone MP6-XT22) and actin (clone ab-5) specific antibodies were purchased from BD Biosciences. Fluorescently labelled CD5 (clone 53-7.3), CD28 (clone E18), CD62 L (clone mel-14), Ki-67 (clone 16A8) specific antibodies and Zombie Aqua (cat. no. 423101) were purchased from Biolegend. Fluorescently labelled anti-IFNγclone 9004) was purchased from Sigma. Anti-phospho-tyrosine residue (clone 4G10) was purchased from Millipore. Anti-phopho-ERK (Thr202/Tyr204) (D13.14.4E, cat. no. 4370) and anti-phospho-S6 (Ser235/236) (D57.2.2E, cat. no. 4858) were purchased from Cell Signalling Technology. Horseradish peroxidase-conjugated anti-mouse antibody was purchased from GE Healthcare (cat. no. NXA931). Horseradish peroxidase-conjugated goat anti-rabbit antibody was purchased from Southern Biotech (cat. no. 4041-05). CD8 T cells isolation kit (130-095-236) and anti-biotin beads (130-090-485) were purchased from Miltenyi and used according to the manufacturer's instructions. ELISA kits for the detection of mouse IL-2 (cat. no. 431001), IFNγ (cat. no. 430801) and TNFα (cat. no. 430901) were purchased from BioLegend. ELISA for mouse IgM (cat. no. 88-50470-22) and IgG (cat. no. 88-50400-22) were purchased from Ebioscience. Recombinant mouse GM-CSF (cat. no. 315-03), IL-2 (212-12) and IL-4 (cat. no. 214-14) were purchased from Peprotech. Carboxyfluorescein succinimidyl ester (CFSE) was purchased from Sigma (cat. no. 21888). Protease inhibitor cocktail was purchased from Roche (cat. no. 11836153001). H-2 L^d^ tetramers were produced and used as described elsewhere [23] or obtained from the NIH tetramer facility. All peptides were produced by the MIT biopolymer facility through standard solid-phase peptide synthesis or purchased from Pepceuticals, UK.

### 4.3. *Toxoplasma gondii* infection

A measure of 1 × 10^5^ sorted CD8^+^ tetramer^+^ cells from pooled spleen and lymph nodes of Rop7-I, -II or -III CD45.2 BALBC/c mice were transferred intravenously into CD45.1 BALB/c recipient. Twenty-four hours after transfer, mice were infected intraperitoneally with 2 × 10^4^ *Toxoplasma* Prugniaud tachyzoites and spleens were harvested 9 days after infection. For *in vitro* re-stimulation, 2 × 10^6^ erythrocytes-depleted splenocytes were incubated for 6 h in the presence of Rop7 peptide (final concentration 1 µg ml^−1^) and a protein transport inhibitor (GolgiPlug, BD Biosciences, cat. no. 555029) in round bottom 96 wells plate in complete RPMI. Intracellular cytokine staining for cytokine was performed after fixation and permeabilization according to the manufacturer's instructions (BD Cytofix/Cytoperm, cat. no. 554722; BD Perm/Wash, cat. no. 554723).

### 4.4. T-cell receptor expression, refolding and affinity measurement

For the Rop7-I, -II and -III TCRs, the α and β chains were cloned individually in the bacterial expression vector pET28a. Residues threonine 46 of the α chain's and serine 57 of the β chain's constant regions were mutated to cysteine to enable non-native disulfide bridge formation within the heterodimer complex [35]. A biotinylation sequence was introduced at the C terminus of the β chain. The chains were separately expressed in *Escherichia coli.* BL21-DE3 (pLysS) and obtained as inclusion bodies and dissolved in 8 M urea, 25 mM MES, 1 mM EDTA, 1 mM DTT at pH 6.0. The proteins were refolded via flash dilution of a mixture of both α and β chains into 1 l refolding buffer composed of 400 mM l-Arginine, 100 mM Tris, 2 mM EDTA, 5 mM reduced glutathione, 0.5 mM oxidized glutathione, pH 8.0 at 4°C to a final protein concentration of 60 mg l^−1^ [36]. The solution was then dialysed with 20 mM Tris, 50 mM NaCl, pH 8.0 to remove denaturants. The resulting TCRs were purified in two steps, first via a size exclusion column (Superdex 200 16/60) and subsequently bound to an anion exchange column (MonoQ 5/50 GL) and eluted with a linear gradient of 0–400 mM NaCl in 20 mM Tris, pH 8.0. The purified TCRs were stored in 20 mM Tris, 50 mM NaCl at −80°C. IPA**A**AGRFF-, IPA**F**AGRFF- and IPA**N**AGRFF-loaded H-2 L^d^ complexes were similarly obtained by established refolding methods [37]. All SPR experiments were conducted on a BIAcore 3000 instrument at 25°C. Soluble streptavidin was first coupled to a CM5 chip (GE Healthcare Life Sciences) by amine coupling following the manufacturer's instructions. For each experiment, approximately 500 resonance units of biotinylated TCR monomers were subsequently immobilized. A flow cell without any TCRs bound served as control for each experiment. The H-2 L^d^ peptide-loaded complexes were directed towards both cells at a flow rate of 20 µl min^−1^, with concentrations ranging from 1 to 200 µM. The final response was calculated after subtracting the control cell response. All experiments were conducted at least in duplicates. Equilibrium *K*_d_ values were calculated by nonlinear curve fitting of background-subtracted data using PRISM software.

### 4.5. *In vitro* stimulation assay

A measure of 1–2 × 10^5^ sorted CD4^+^ or CD8^+^ tetramer^+^ cells from pooled spleen and lymph nodes of Rop7-I, -II or -III mice were stimulated in complete RPMI in 96-well flat bottom plates in the following conditions. Anti-CD3/28: plate-bound anti-CD3 (clone 17A2) and anti-CD28 (clone 37.51) at indicated concentrations in flat bottom 96-well plates in the presence of 10 ng ml^–1^ recombinant mouse IL-2 when specified. BMDC: dendritic cells differentiated from bone marrow progenitors for 5–7 days in GM-CSF and IL-4 containing medium loaded with Rop7 peptide (final concentration 1 or 0.01 µg ml^−1^). When indicated, 10–20 × 10^6^ cells per millilitre were labelled in PBS 2.5 µM CFSE for 2 min at room temperature.

### 4.6. Cytokine secretion measurements

A measure of 1–2 × 10^5^ sorted CD4^+^ or CD8^+^ tetramers^+^ cells from pooled spleen and lymph nodes of Rop7-I, -II or -III mice were stimulated in complete RPMI in 96-well round bottom plates together with 0.1–0.5 × 10^5^ Rop7 peptide-loaded BMDC (final concentration 1 or 0.01 µg ml^−1^). Cytokine concentrations were measured by ELISA according to the manufacturer's instructions or by multiplex assay (Eve Technologies, Calgary, Alberta, Canada).

### 4.7. *In vitro* cytotoxicity assay

A measure of 1–2 × 10^5^ sorted CD4^+^ or CD8^+^ tetramers^+^ cells from pooled spleen and lymph nodes of Rop7-I, -II or -III mice were stimulated in complete RPMI in 96-well round bottom plates together with 0.5 × 10^5^ Rop7 peptide-loaded BMDC (final concentration 1 µg ml^−1^). After 72 h, cells were harvested and incubated with 10^5^ total cells of a 1 to 1 mixture of CFSE-labelled, Rop7 peptide-loaded CD45.1 BALB/c splenocytes (final concentration 1 µg ml^−1^) and control, CFSE-labelled, DMSO-treated CD45.2 BALB/c splenocytes in 96-well round bottom plates. After 16–20 h the ratio of living (propidium iodide negative) CD45.1 versus CD45.2 cells was determined by flow cytometry.

### 4.8. *In vitro* tetramer stimulation

Total CD8 T cells were purified from pooled lymph nodes and erythrocytes-depleted spleens of Rop7-I, -II or -III mice, homozygous for α and β chain by negative selection using magnetic beads according to the manufacturer's instructions. Two to five million tetramer^+^ cells were stimulated with 13 µg Rop7 peptide-loaded H-2 L^d^ tetramers in 50 µl complete RPMI at 37°C. Cells were then washed with ice cold PBS and lysed in 20 mM Tris–HCl, pH 7, 150 mM NaCl, 5 mM MgCl_2_, 5 mM Na_3_VO_4_, 10 mM NaF, 0.5% NP40 supplemented with protease inhibitor cocktail (Roche). Total lysates were run on a 12% acrylamide SDS-PAGE gel. After transfer to a nitrocellulose membrane, phosphorylation events were monitored by blotting using phospho-tyrosine, phospho-ERK or phospho-S6 specific antibodies.

### 4.9. *In vivo* luminescence measurement

An amount of 8 × 10^4^ sorted CD8^+^ tetramer^+^ cells from pooled spleens and lymph nodes of Rop7-I, -II or -III mice were transferred intravenously into BALB/c recipients. Twenty-four hours after transfer, mice were infected intraperitoneally with 5 × 10^4^ *Toxoplasma* Prugniaud tachyzoites. For *in vivo* imaging, mice were injected i.p. with 3 mg firefly d-luciferin (Perkin Elmer, Waltham, MA, USA), left for 10 min and imaged with an IVIS Spectrum-bioluminescent and fluorescent imaging system (Xenogen Corporation, Caliper Life Sciences, Hopkinton, MA, USA) under isoflurane anaesthesia (Abbott, Chicago, IL, USA) on day 4–7 post-infection.

### 4.10. RNA-sequencing analysis

Single-cell suspensions of unstimulated splenocytes from Rop7-I, -II or -III mice were incubated in RPMI medium 1640 supplemented with 10% fetal bovine serum, l-glutamine, penicillin/streptomycin, β-mercaptoethanol and recombinant mouse IL-2 (10 ng ml^−1^). Cells were incubated overnight at 37°C and 5% CO_2_. CD3^+^ CD8^+^ H-2 L^d^-Rop7^+^ cells were sorted, maintained at 4°C and purity determined to be 90–95%. RNA was isolated using Trizol and the RNeasy Micro-Kit (Qiagen). A total of 200 ng of RNA was used to prepare the RNA library using TruSeq mRNA Library Prep Kit v2 (Illumina) according to the manufacturer's recommendations. RNA sequencing was performed on the Illumina HiSeq 2500 and typically generated approximately 25 million 100 bp non-strand-specific single-end reads per sample. The RSEM package (v. 1.2.11) [38] was used for the alignment and subsequent gene-level counting of the sequenced reads relative to mm10 RefSeq genes downloaded from the UCSC Table Browser [39] on 27 May 2015. Differential expression analysis between the triplicate groups was performed with DESeq2 (v. 1.8.1) [40] after removal of genes with a maximum transcript per million (TPM) value of 1 across all samples in the experiment. Significant expression differences were identified at an FDR threshold of 0.01. Gene set enrichment analysis was performed by Gene Ontology Biological processes using GeneGo MetaCore (https://portal.genego.com/). Pathway analysis was performed using IPA software to demonstrate the biological effect of differentially expressed genes on cell cycle progression. All raw RNASeq sequence data and per sample TPM counts generated by RSEM can be accessed with GEO accession GSE88996.

## Supplementary Material

Supplementary Figure 1.

## Supplementary Material

Supplementary Figure 2.

## Supplementary Material

Supplementary Figure 3.

## Supplementary Material

Supplementary Figure 4.

## Supplementary Material

Supplementary Table 1.

## Supplementary Material

Supplementary Table 2.

## Data accessibility

All raw RNASeq sequence data and per sample TPM counts generated by RSEM can be accessed with GEO accession GSE88996.
