# Supplementary figures and images for: Peripheral self-reactivity regulates antigen-specific CD8 T-cell responses and cell division under physiological conditions
Source: Open Biol. 2016 Nov 23;6(11):160293. doi: 10.1098/rsob.160293 (PMC5133449; doi:10.1098/rsob.160293)

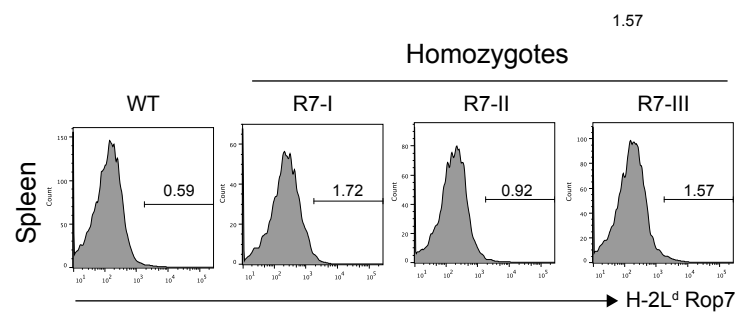

Supplement: Supplementary Figure 1. [file rsob160293supp1.pdf]

### Transfer of CD8+/tet+ ROP7 TN T cells

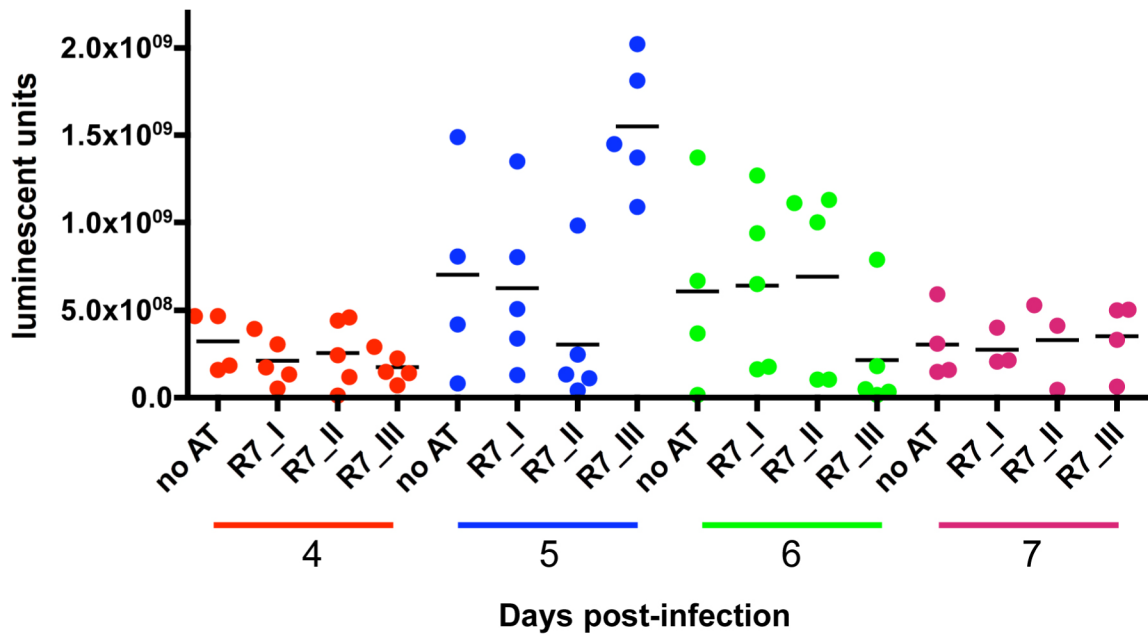

Supplement: Supplementary Figure 2. [file rsob160293supp2.pdf]

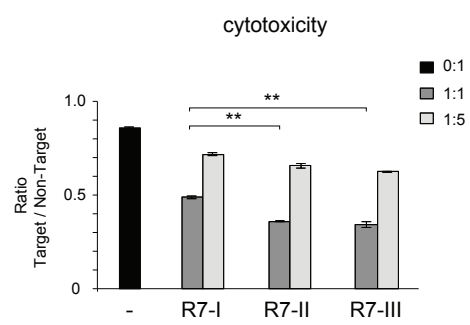

Supplement: Supplementary Figure 3. [file rsob160293supp3.pdf]

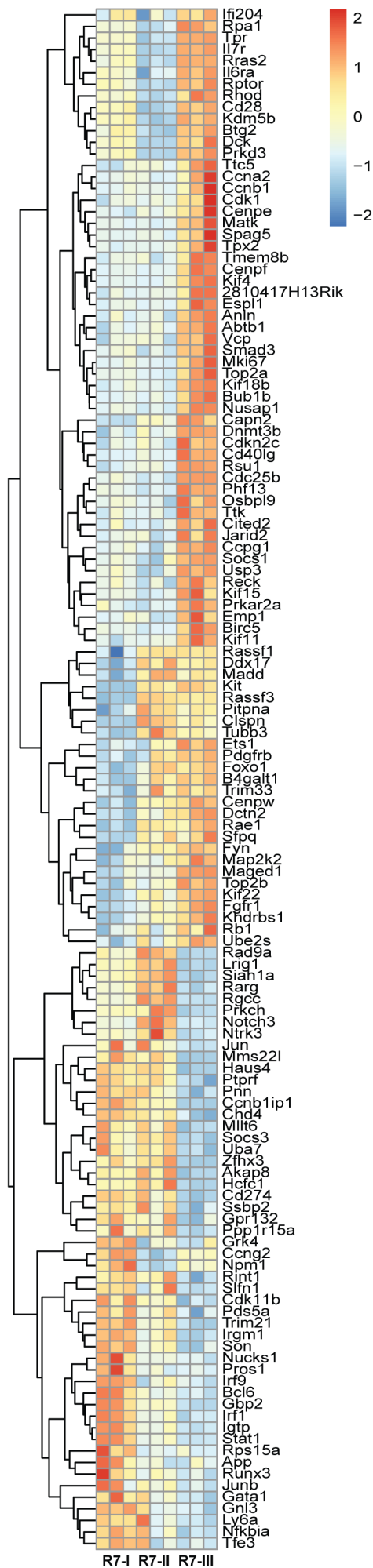

Supplement: Supplementary Figure 4. [file rsob160293supp4.pdf]
