# Supplementary material for: Peripheral self-reactivity regulates antigen-specific CD8 T-cell responses and cell division under physiological conditions
Source: Open Biol. 2016 Nov 23;6(11):160293. doi: 10.1098/rsob.160293 (PMC5133449; doi:10.1098/rsob.160293)
Supplement: Supplementary Table 1. [file rsob160293supp5.pdf]

#	gene	fold up	FDR
1	Scg5	13.80	4.41E-234
2	Col6a4	5.99	9.66E-61
3	Dapl1	5.28	5.38E-77
4	Msgn1	4.40	3.87E-43
5	Smc6	4.33	4.51E-258
6	Cpne4	4.06	1.80E-35
7	Ubash3b	3.93	3.35E-54
8	Ptgfrn	3.82	1.18E-46
9	Ptger2	3.54	5.74E-28
10	Pkp4	3.47	8.17E-68
11	Cd163l1	3.44	1.52E-27
12	Cd5	3.41	1.00E-48
13	Acpp	3.37	3.82E-111
14	Top2a	3.37	1.21E-26
15	Mki67	3.17	3.83E-25
16	Itga9	3.13	3.53E-27
17	Cd101	2.92	8.48E-28
18	A430093F15Rik	2.91	8.76E-21
19	St8sia6	2.89	6.54E-41
20	Slc43a1	2.84	3.49E-19
21	Ppp1r3e	2.70	1.26E-18
22	Rpgrip1	2.67	8.17E-26
23	Sall2	2.63	1.83E-22
24	Phldb3	2.61	3.30E-33
25	Ank	2.61	7.68E-76
26	Ccl5	2.60	4.04E-16
27	Itga6	2.59	1.07E-72
28	Gch1	2.56	1.86E-27
29	Nacc2	2.50	3.23E-18
30	Cenpf	2.48	6.14E-15
31	Cd6	2.44	1.52E-17
32	Rhod	2.40	7.04E-14
33	Lysmd2	2.40	1.75E-14
34	Fbxl3	2.39	7.60E-119
35	Camk2b	2.38	1.60E-13
36	Mfap1a	2.36	8.69E-52
37	St3gal6	2.34	2.95E-13
38	Dap	2.33	5.96E-89
39	Il7r	2.33	1.09E-121
40	Tex9	2.31	3.25E-23
41	Pltp	2.30	5.82E-17
42	Ltk	2.29	8.67E-15
43	Tbc1d4	2.28	1.28E-53
44	Nusap1	2.26	6.92E-12
45	Bbc3	2.26	1.66E-35
46	Runx2	2.25	3.63E-27
47	Adk	2.22	8.56E-38
48	Aspm	2.21	2.94E-11
49	Arhgef3	2.21	7.60E-104
50	Igf2bp3	2.19	3.33E-13
51	Per2	2.17	7.96E-11
52	Fam221a	2.16	4.61E-13
53	Ybx3	2.15	1.06E-11
54	Gm5538	2.14	1.33E-12
55	Ccdc88a	2.13	1.78E-12
56	Mgat5	2.11	4.13E-21
57	Pde5a	2.10	1.20E-25
58	Fam219a	2.10	1.31E-16
59	Dusp4	2.09	3.76E-10
60	Rcn1	2.06	1.57E-15
61	Mllt4	2.05	2.73E-20
62	Xdh	2.05	2.54E-10
63	Hip1r	2.03	1.76E-10
64	Hs3st3b1	2.02	1.88E-12
65	Cdc42ep3	2.01	2.93E-20
66	Sidt1	2.01	2.37E-45
67	Pear1	2.00	5.09E-12

#	gene	fold down	FDR
1	Slc6a19	-9.64	1.43E-222
2	Abhd14b	-6.35	2.45E-60
3	Pdk1	-5.51	0
4	Crip1	-4.63	3.98E-52
5	Tmie	-4.47	8.25E-91
6	Notch3	-4.04	1.46E-44
7	Zfp862-ps	-3.38	4.69E-38
8	Cmtm7	-3.22	8.25E-91
9	Pcsk1	-3.12	3.74E-24
10	Frmpd1	-3.12	9.33E-23
11	Rgcc	-3.11	6.50E-92
12	Als2cl	-3.05	4.10E-119
13	Tdgf1	-2.94	3.75E-23
14	Dok2	-2.83	3.43E-98
15	Atg14	-2.78	3.02E-91
16	Atp6v1d	-2.77	1.54E-22
17	Kcnj16	-2.75	1.56E-18
18	Repin1	-2.74	5.89E-18
19	Abhd14a	-2.71	1.67E-17
20	Pttg1	-2.70	5.42E-20
21	Gm16907	-2.69	1.38E-17
22	Abca1	-2.68	1.98E-17
23	Gpr114	-2.64	2.32E-21
24	Prrg4	-2.57	1.04E-16
25	Nfam1	-2.44	7.21E-19
26	Pcbp4	-2.35	2.11E-15
27	Dtx1	-2.35	7.81E-25
28	Gcm1	-2.34	5.35E-13
29	Sema4f	-2.33	7.18E-29
30	Samd4	-2.32	8.21E-14
31	Ntrk3	-2.31	1.03E-13
32	Rnf144a	-2.30	1.86E-12
33	Reep1	-2.28	1.78E-12
34	Itgae	-2.27	1.51E-36
35	Mns1	-2.25	4.28E-12
36	Tln2	-2.22	8.15E-12
37	Gpr97	-2.19	4.63E-13
38	Dstyk	-2.17	7.24E-19
39	Sipa1l2	-2.15	5.13E-11
40	Ly6e	-2.14	1.16E-10
41	Zfhx3	-2.09	1.96E-10
42	C920006O11Rik	-2.08	5.69E-10
43	Gsg2	-2.08	1.04E-20
44	Rgs3	-2.05	2.91E-47
45	Ksr2	-2.04	2.09E-10
46	D930028M14Rik	-2.03	1.24E-14
47	Galnt9	-2.03	2.30E-11
48	Lynx1	-2.02	5.19E-09
