# Supplementary material for: Peripheral self-reactivity regulates antigen-specific CD8 T-cell responses and cell division under physiological conditions
Source: Open Biol. 2016 Nov 23;6(11):160293. doi: 10.1098/rsob.160293 (PMC5133449; doi:10.1098/rsob.160293)
Supplement: Supplementary Table 2. [file rsob160293supp6.pdf]

| # | Biological Processes | in data/total | FDR |
|---|---|---|---|
| 1 | Cell cycle_Mitosis | 48/179 | 5.01E-07 |
| 2 | Cell adhesion_Leucocyte chemotaxis | 48/205 | 1.95E-05 |
| 3 | Cell cycle_G2-M | 48/206 | 1.95E-05 |
| 4 | Cytoskeleton_Regulation of cytoskeleton rearrangement | 43/183 | 4.71E-05 |
| 5 | Cell adhesion_Integrin-mediated cell-matrix adhesion | 45/214 | 4.17E-04 |
| 6 | Cell cycle_Core | 29/115 | 4.17E-04 |
| 7 | Development_Hemopoiesis, Erythropoietin pathway | 32/136 | 6.06E-04 |
| 8 | Immune response_TCR signaling | 37/174 | 1.37E-03 |
| 9 | Proliferation_Lymphocyte proliferation | 42/209 | 1.60E-03 |
| 10 | Immune response_Phagosome in antigen presentation | 46/243 | 2.76E-03 |
| 11 | Signal Transduction_Cholecystokinin signaling | 25/106 | 2.76E-03 |
| 12 | Cell adhesion_Integrin priming | 25/110 | 4.47E-03 |
| 13 | Cell cycle_G1-S Growth factor regulation | 38/195 | 4.47E-03 |
| 14 | Cell cycle_S phase | 31/149 | 4.47E-03 |
| 15 | Cell adhesion_Platelet aggregation | 32/158 | 5.58E-03 |
| 16 | Cytoskeleton_Spindle microtubules | 24/109 | 7.42E-03 |
| 17 | Cell cycle_Meiosis | 23/106 | 1.08E-02 |
| 18 | Proliferation_Positive regulation cell proliferation | 40/221 | 1.08E-02 |
| 19 | Signal transduction_NOTCH signaling | 42/236 | 1.10E-02 |
| 20 | Development_Neurogenesis_Axonal guidance | 41/230 | 1.15E-02 |
| 21 | Cell adhesion_Attractive and repulsive receptors | 33/175 | 1.17E-02 |
| 22 | Apoptosis_Apoptotic mitochondria | 18/77 | 1.17E-02 |
